# Participatory Methodologies for Addressing School Bullying: An Overview and Methodological Guidelines

**DOI:** 10.3390/children13020214

**Published:** 2026-01-31

**Authors:** Manuel Montañés-Serrano, Iving Zelaya-Perdomo, Esteban A. Ramos Muslera

**Affiliations:** 1 Department of Sociology and Social Work, University of Valladolid, 40005 Segovia, Spain; mms@soc.uva.es; 2 Institute for Research in the Humanities, National Autonomous University of Honduras, Tegucigalpa 11101, Honduras; 3 University Institute for Democracy, Peace and Security, National Autonomous University of Honduras, Tegucigalpa 11101, Honduras; esteban.ramos@unah.edu.hn

**Keywords:** adolescence, bullying, childhood, identity, participatory methodologies, peacebuilding, networks, sociopraxis

## Abstract

**Highlights:**

**What are the main findings?**

- Bullying is a relational phenomenon that involves multiple group networks, rather than a simple dyadic interaction between victim and perpetrator; in bullying violence functions as a mechanism for constructing group identity, defining an “us” through the systematic non-recognition of certain others.
- Traditional approaches to bullying—centered on studying prevalence rates and identifying individual traits—do not adequately explain its causes or how the phenomenon unfolds in specific contexts.

**What is the implication of the main finding?**

- Preventing bullying or stopping it once it has emerged requires undermining the social support base that sustains it and fostering a shared stance in favor of diversity and against heteronormative and exclusionary norms.
- A participatory process involving the entire educational community in the design and implementation of a School Coexistence Plan—guided by the Participatory Construction of Peaceful Coexistence method—offers an appropriate, efficient, and effective pathway for transforming these dynamics.

**Abstract:**

Bullying is not a dyadic interaction between victim and perpetrator, but a relational phenomenon involving multiple group networks: those who exercise physical, psychological, or symbolic violence; those who encourage it; those who suffer it; and those who, while aware of it, remain on the sidelines. Preventing bullying, or stopping it once it emerges, requires undermining the support base that sustains it: no one should play the role of cheerleader, and those who remain passive must become involved in defending those targeted. It is also necessary to foster in those who are bullied the strength and capacity to confront the situation. From a Freirean perspective, this implies weaving alliances between those who are kindred and those who are different, and even with outsiders, to oppose those who act antagonistically. Such a task demands debate, reflection, and the collective formulation of measures among the diverse group realities in schools, given that bullying is grounded in the refusal to recognize certain others as part of “us”, though we are all “others” to one another. This article sets out arguments for the need to address these diverse group realities and presents the phases and main contents of a participatory process for designing and implementing a School Coexistence Plan, drawing on the Participatory Construction of Peaceful Coexistence method as a framework for addressing bullying.

## 1. Introduction

Bullying is conceived as a specific form of peer violence, distinct from isolated aggressive acts, and is commonly experienced in childhood and adolescence [1]. Although there are some relatively stable elements in the definition of the term bullying, its conceptualization remains contested [2]. In academic settings, the different conceptualizations are shaped by the disciplinary field to which the concept is affiliated, as well as by the sociocultural context, leading to variations that depend on the specific environment in which the phenomenon is studied [3].

Drawing on different existing definitions, bullying has been described as an aggressive, repetitive, and intentional behavior grounded in a power imbalance and directed toward a person or group that cannot easily defend themselves [4]. In a similar vein, bullying has also been defined as direct or indirect aggression, intentional and repeated in nature, that entails a power imbalance between those who perpetrate it and the victims [5]. These conceptualizations encompass physical, social, symbolic, and digital forms of aggression. In a related line, bullying has been defined by UNESCO [6] as a harmful social process characterized by an imbalance of power driven by social and institutional norms. It is often repeated and manifests as unwanted interpersonal behavior among students or school staff that causes physical, social, and emotional harm to targeted individuals or groups, as well as to the school community as a whole.

Academic and institutional definitions often contrast with those developed by children and young people [3,7]. Children’s conceptions may include isolated episodes or situations in which the criteria of repetition, power imbalance, and intentionality are not necessarily met [3,7]. In this sense, the boundary between bullying and other forms of aggression is contested, especially when the perceptions of children themselves are taken into account.

The etiology of bullying is not limited to individual traits; it is a multi-causal phenomenon shaped by group dynamics, family contexts, institutional cultures, and socio-technical environments. Previous research has shown that bullying is a complex phenomenon that undermines well-being, academic performance, and the long-term development of children and young people [8]. It has also been argued that those who bully often seek to gain status within their peer group and that bullying is grounded in group role dynamics, including the roles of aggressors, assistants, reinforcers, defenders, and bystanders [9]. From this perspective, bullying practices are mediated by group structure and norms rather than stemming only from isolated personality traits. Other studies emphasize that the emergence and persistence of bullying largely depend on the classroom climate, the explicit and implicit norms that are established, and the way in which parents and caregivers react to aggression [10]. In a similar vein, it has been argued that addressing bullying requires both adults and youth to work on shared agendas that take into account the different definitions of the phenomenon, its effects, and the support mechanisms available [3]. The interaction networks in which students, institutional actors, and families participate therefore need to be considered when approaching bullying from an intergenerational perspective.

Research on social and family contexts underlines their importance in preventing adolescent risk factors that create conditions in which peer aggression becomes normalized, such as domestic violence, substance use, and low-supervision parenting styles [11]. Further evidence indicates that the proliferation of physical, verbal, and cyber abuse has led education policymakers to seek more holistic prevention approaches that address policy design, teacher training, the availability of psychosocial support resources, reporting mechanisms, and institutional culture [8].

Likewise, several studies emphasize that bullying is embedded in broader patterns of inequality and violence, arguing that aggressive behavior is intertwined with prevailing gender norms, cultural forms of violence, socioeconomic inequalities, and existing mechanisms for conflict resolution [12,13,14,15].

UNESCO’s whole-education approach provides an additional conceptual lens that is consistent with this relational and multi-causal understanding of bullying. Rather than situating bullying solely within the boundaries of individual schools or interpersonal dyads, UNESCO [6] frames it as a phenomenon that unfolds within interconnected education, technological, and societal systems, and therefore must be addressed through coordinated action across these levels. From this perspective, effective responses require strong political leadership, adequate legal and policy frameworks, and the development of a caring school climate through curriculum, teaching, and classroom management oriented towards inclusion and non-violence. They also demand safe physical and psychosocial environments, clear reporting mechanisms and referral pathways, and systematic training and support for school staff. Central to this approach is the active participation and empowerment of students, alongside the involvement of families and other community stakeholders, and the establishment of partnerships with other government sectors, civil society, academia, and digital platforms. Monitoring bullying and evaluating the effectiveness of interventions are conceived as integral components rather than ancillary tasks, reinforcing the idea that the nine elements of the whole-education approach should operate as an integrated system, not as a “menu” from which isolated measures are selected [6].

Traditional approaches to studying, preventing, and managing bullying have focused primarily on calculating prevalence rates and identifying individual traits, which do not adequately account for its causes or for how the phenomenon unfolds in specific contexts [3]. Higher levels of participation by those directly involved have been shown to lead to a more comprehensive and situated understanding of bullying [3]. Extending this critique of what has been described as an adult-centric perspective [16], other contributions maintain that it is necessary to use methodologies that enable students to express, from their own standpoint and in their own language, the seriousness of different bullying situations, taking into account the context, the repetition of events, and the responses of parents and caregivers [5,16]. This is not simply a matter of asking students questions, but of incorporating mechanisms that allow the different actors and networks involved to construct and articulate their own interpretive frameworks and proposals for action.

In this vein, a participatory action research methodology has been proposed by O’Brien and Doyle to address school bullying, structured in two stages [4]. In an initial exploratory phase, an online questionnaire is administered to the school community to identify core bullying issues. Subsequently, iterative work cycles are developed in which a group of students, together with teacher advisers, define the research question, the mechanisms for data collection, and the ways in which the results will be communicated [4]. This process entails an ongoing negotiation of power relations between young people and adults, generating cycles of reflection and continuous readjustment.

In a similar direction, a participatory exercise has been documented by O’Brien and Dadswell in which a group of young people who had dropped out of school, together with educational and therapeutic support staff, co-constructed the concept of bullying and participated in a series of focus groups to define the problems and identify possible forms of support to mitigate it [17]. In the systematization of the INKLA project implemented in Poland, a shift in agency towards the student body is reported, as secondary school students, supported by university researchers and students, initiated a youth participatory action research process in which pairs of student-researchers interviewed classmates and teachers about intra-group relations, bullying, and peer group exclusion. These inquiries fed into research-based collective actions aimed at preventing bullying, strengthening supportive peer groups, and improving everyday school life, showing that student voice can inform and even reshape a school’s anti-bullying policy when responsibility for prevention is genuinely shared with young people who are recognized and treated as agents of change [10].

The development of formal education programs on bullying has also been approached through co-design work with early childhood teachers, using interviews, discussions, and Q-sort techniques to identify the problem, explore its causes, and design an early childhood education and prevention program adapted to the Australian school context [18]. Similarly, participatory youth evaluation has been used to review bullying prevention programs prior to their implementation in specific contexts, bringing together young leaders to examine curricula and prioritize those activities with the greatest potential impact [19].

The field of cyberbullying provides additional elements for the application of participatory approaches, in this case from the design of technologies. Co-design workshops have been implemented to imagine and prototype support services for the safe reporting of cyberbullying experiences [20]. These approaches do not limit themselves to consulting users but rather incorporate their experiences as a central criterion for defining the requirements, functionalities, and interaction flows of future systems [20]. Other studies have developed processes in which, over several sessions, high school students share their experiences with cyberbullying and formulate potential solutions using low-fidelity prototypes [21]. The use of participatory designs to address cyberbullying is understood not as a mere add-on, but as a process through which priorities, languages, and technological mediations are redefined based on the experiences of those who live with the phenomenon on a daily basis [22].

Bullying has been studied from various theoretical perspectives and through multiple methodological proposals for its analysis, prevention, and treatment. Among these, approaches that incorporate participatory methodologies are particularly relevant, as they seek to foster a deeper understanding of the phenomenon and a more effective transformation of the dynamics that sustain it. Taking participatory methodologies in Peace and Conflict Studies as a reference, this article explores a participatory perspective that uses such methodologies to promote the analysis, collective reflection, and transformation of bullying, taking into account its relational nature and the specific school contexts in which it occurs. First, the approach from which the phenomenon is understood, anchored in a sociopraxical perspective, is presented. Second, the methodological strategy designed for its participatory transformation is set out. Third, the dialogue between the conceptions outlined in this introduction and the proposed method for addressing bullying is discussed. Finally, some conclusions are offered, conceived as provisional and open-ended, which aim to contribute, in a spiral fashion, to the construction of theoretical, conceptual, and methodological frameworks that help to address bullying within scientific and school communities, as well as in society as a whole.

## 2. A Methodological Strategy for the Participatory Transformation of Bullying

Bullying, like any other social reality, is not a given fact but a socially constructed phenomenon. A situation that, for some, is seen as intimidation and violence that generates pain, worry, fear, and anxiety, may be perceived by others as an act of reaffirming their group identity by mocking those they consider to “deserve” such treatment because of their physical, social, cultural, or other characteristics.

The forms of violence deployed in bullying can be understood within what has been termed a culture of violence, in which people act violently because they have been socialized to view violence as a legitimate mean and mode of meeting their needs and/or dealing with present or foreseeable conflicts. In this perspective, individuals resort to aggression in order not to be become victims of the violence of others—echoing the common saying that there is no better defense than attack. However, bullying is not only carried out by children and young people who have been socialized in explicitly violent environments. Those who are socialized in non-violent environments may also bully other children and adolescents.

In bullying, violence is not only a means of extortion, theft, or intimidation; it is also a way of humiliating the person who is targeted. Bullying becomes a mechanism for constructing group identity based on the non-recognition of the other. Systematically turning the other into an object of ridicule, criticism, contempt, and psychological—and sometimes physical—violence is a way of consolidating and reinforcing a sense of belonging among those who participate in or tolerate such practices.

Harassment is thus grounded in the refusal to recognize the other as part of “us”, whether because they are short or tall, obese or thin, have different physical, psychological, or cognitive abilities, identify as homosexual, transgender, asexual, or intersex, belong to a stigmatized ethnic group, are migrants, female, or poor, or do not dress or style their hair in ways deemed appropriate. In all these cases, difference becomes a pretext for exclusion and aggression.

This identity construction does not involve only those who bully and those who are bullied. As in any socially constructed reality, multiple networks and groups are implicated: those who directly exercise physical, psychological, or symbolic violence; those who encourage or celebrate these actions; those who suffer them; and those who are aware of what is happening and remain on the sidelines, whether out of fear of becoming the next targets, because they consider that those who are victimized deserve it in some. way, or because, as long as they are not directly affected, they do not see it as their concern and, therefore not as their problem. In addition, the existence of online networks means that bullying extends beyond the physical boundaries of the school.

Resorting to school regulations, which set out rights and duties, offenses, and penalties, is insufficient to prevent bullying. This is not only because, as noted above, bullying transcends the school walls, but also because the mere existence of disciplinary rules does guarantee they will be respected. Even the use of sanctions can generate an undesirable effect, since their application does not necessarily lead to the disappearance of bullying, and may aggravate it: the person who has been bullied may suffer reprisals from the sanctions student or group and/or those close to them, who hold the victim responsible for the measures adopted by the school administration. The enforcement of rules that have been drafted without the participation of those to whom they are applied may also provoke rejection—and corresponding reactions—on the part of the sanctioned person and others who identify with them. If sanctions can thus generate further tension, it might be assumed that activating school mediation mechanisms could offer a way to address bullying.

Those who advocate for this option should be aware that, although school mediation may contribute in some specific situations, it is unlikely to be sufficient or to generate sustainable transformations when used as the main mechanism for addressing bullying. Among other reasons, mediation requires voluntary participation and presupposes a degree of symmetry in power between the parties that is difficult to achieve in relationships marked by power imbalances. It must also be borne in mind that relying primarily on the aggressor’s goodwill is unlikely, on its own, to bring harassment to an end or to dismantle the relational structures that sustain it.

Preventing bullying, or stopping it once it has emerged, requires undermining the support base that sustains the bully. It is necessary to foster an opinion climate that is favorable to diversity and critical of heteronormative models. This involves working towards the construction of a sense of “us” that recognizes the other, because we are all “others” to one another—even to ourselves—since, as Heraclitus reminded us, in a reality that is constantly changing it is not possible to step into the same river twice.

An opinion climate that affirms otherness can reduce the environment of complicity that surrounds the bully, weaken or even eliminate those who cheer them on, encourage bystanders to become involved in defending those who are targeted, and help those who are bullied to find the strength and self-esteem to confront harassment. Unfortunately, in many cases, those who experience bullying are also subjected to symbolic violence [23], which leads them to believe that they deserve the attacks because of who they are or how they behave.

The creation and consolidation of climate favorable to diversity can be significantly strengthened by implementing a participatory process involving the entire educational community in the design of a School Coexistence Plan. The very act of taking part in the formulation of rules, as well as in the bodies responsible for monitoring and enforcing them, constitutes a practice rooted in respect for, recognition of, and commitment to the defense of social diversity.

### 2.1. The Method for the Participatory Construction of Peaceful Coexistence

The Participatory Construction of Peaceful Coexistence method [24,25,26], designed to promote the collective construction of peace and anchored in the sociopraxical perspective of Transformative Peace [27,28,29], is used here as a strategy for reconfiguring the relational structures that sustain bullying. Drawing on proposals that advocate weaving together related and different networks in order to engage those who are alienated and indifferent, while isolating and/or transforming the behavior of those who oppose change [30], the method seeks to foster the broadest and densest possible constellation of actions [29]. By defending diversity and promoting an “us” based on the recognition of the other, these actions aim to prevent bullying or to confront it when it has already emerged.

**Figure 1:**
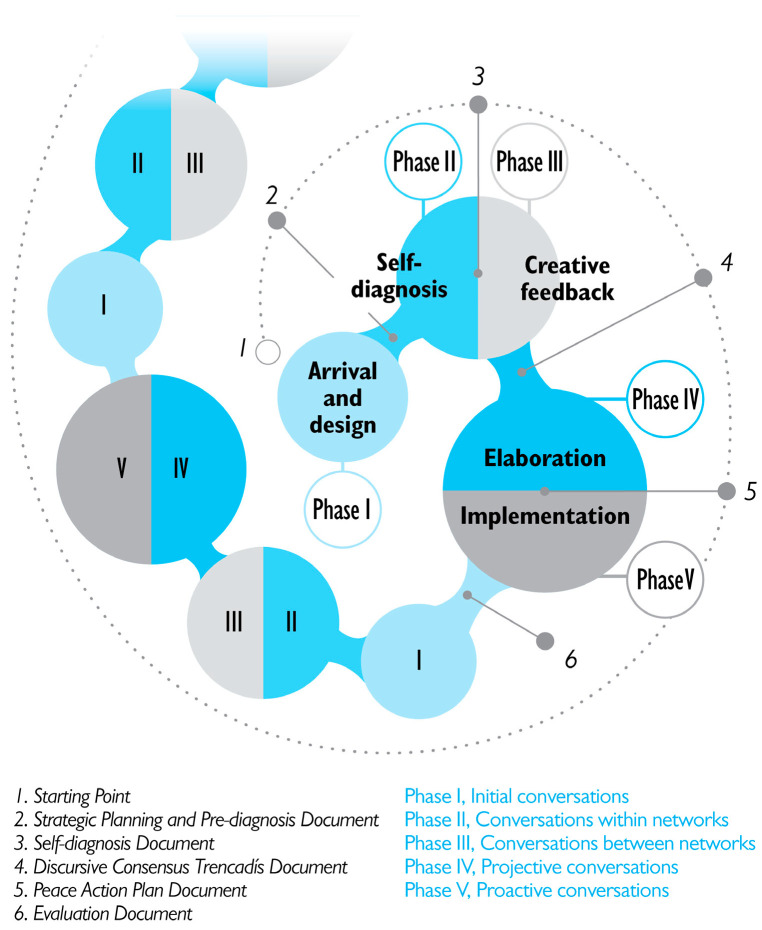
Methodological Process of the Participatory Construction of Peaceful Coexistence [24].

As shown in Figure 1, the methodological process unfolds as a dialogical sequence of individual and collective action–reflection–action structured in five phases: Phase I, Initial conversations; Phase II, Conversations within networks; Phase III, Conversations between networks; Phase IV, Projective conversations; and Phase V, Proactive conversations.

The phases are implemented as an expansive spiral that promotes the transformation of attitudes, positions, and behaviors across the different networks linked to bullying. Through successive and, at times, concurrent spaces for analysis, reflection, and dialogue within and between networks, the methodological process seeks to re-unify feeling–thinking [31] to diagnose and intervene in specific conflict situations through the participatory construction and implementation of a Plan for Peaceful School Coexistence. The proposed method functions as a model of social dynamization facilitated by a team whose insertion in the context should foster both the development of a shared self-diagnosis of bullying and its impacts and feeling–thinking reflection and collective action to confront the situation and to design convivial, synergistic ways of attending to needs.

To this end, the method calls for the synchronous development of three strategic lines and the implementation of a set of participatory and engaging components, techniques, dynamics, and tools, that combine qualitative and quantitative approaches, namely:

- The strategic line of Research for Transformative Peace, which draws on content and methodological proposals for action research developed in Latin American participatory traditions [32,33,34,35], with the aim of investigating and characterizing the phenomenon of bullying and its impacts;
- The strategic line of Education for Transformative Peace [36], which seeks to promote the collective construction of knowledge, sensitivity, and the reflective feeling–thinking necessary to foster personal and social transformation, in a Freirean emancipatory key [30,37];
- The strategic line of Direct Action for Transformative Peace, which adapts contributions from Peace and Conflict Studies to a participatory approach geared towards the effective construction of peace and the nonviolent transformation of conflicts in a Gandhian key [38], drawing on approaches such as the Transcend Method [39], the Integrated Conceptual Framework [40], and Mediation for Peace [41].

In each phase, the preparation of working documents is envisaged, serving a dual purpose. First, they summarize and systematize the findings, lessons learned, and agreements reached, thus operating as closing or concluding points. Second, they function as starting points for the subsequent phase, providing continuity and coherence to the process and helping to avoid dispersion or the endless prolongation of debates.

The methodological strategy to be followed depends on the initial scope defined for the process, with the possibility of expanding it in line with the degree of participation and commitment of the different actors in the educational community and the real possibilities offered by the context. The initial scope may range from a comprehensive exploration of the phenomenon of bullying and its impact on coexistence and governance in the educational center and its sociocultural environment, to a focus on a particular case. The process may be adapted to a specific type of bullying that affects the entire educational community, or developed as an intervention in a specific group, level, or grade [34]. Regardless of the scope defined, the methodological design must assume the phenomenon as a “total social fact” [42], promoting dynamics of action–reflection–action within school networks. Even when the process focuses on a particular sphere or case, it is necessary to link the problem to broader structural, relational, and cultural factors to ensure a systemic understanding of the phenomenon and to promote sustainable processes of transformation.

The main methodological content of the five phases that structure the process is outlined below, with reference to the components, techniques, dynamics, and tools associated with each of them.

#### 2.1.1. Phase I: Initial Conversations

In the first phase of the Participatory Construction of Peaceful Coexistence process, the methodological development of the approach must be planned and its organizational structure defined, implementing a set of components that situate the process in its specific context. The starting point is the creation and integration, within the school, of a technical team responsible for coordinating and guiding the methodological process and for ensuring the proper development of its components. In this first phase, the technical team—ideally composed of people from diverse fields, such as peace education, ethnography, social psychology, communication, community development, sociology, and anthropology, among others, and with strong social skills for interacting with different actors, age groups, and networks—is expected to carry out an internal self-reflection exercise aimed at analyzing its own role as researcher–facilitator of the process. For this purpose, tools such as self-reflection matrices [43] and role-playing or sociodrama dynamics [44] can be used to problematize the team’s own positions, expectations, and modes of involvement. At the same time, the Education for Transformative Peace strategy is initiated through an internal analysis of existing capacities and limitations in terms of knowledge, abilities, and skills, with the aim of strengthening the team’s competences in line with the particularities of the context.

It is also necessary to establish formal support mechanisms through the creation of a Monitoring Committee [43,45], in order to facilitate coordination between the technical team and the institutions, the school management team, parents’ associations, and student associations, where they exist. As its name suggests, this committee is responsible for the continuous monitoring and supervision of the process, with the search for consensus guiding its work and coordination among the different actors that comprise it being its main value. For this purpose, the diversity of needs, demands, and motivations of the different actors represented in the committee must be identified, so as to reach a Trust Agreement [46] based on an Initial Negotiation [43,45], in which the basic operating rules and the commitment of all actors to the participatory logic of the process are formalized.

The integration of the technical team into the educational community is a key component of the methodological process at this stage. In this regard, it is advisable to establish contact with as many of the networks that make up the educational community as possible, including actors from different student groups, teaching staff, and parents. The creation of a Network Partner System is useful for this purpose, as it promotes the organization and systematization of these relationships [46]. It is also necessary to form a Steering Group, composed of the technical team and those actors who are most willing to collaborate in the day-to-day development of the process [43,45]. While the main function of the Network Partner System is to keep the technical team in contact with the educational community networks—updating them on their demands, interests, concerns, and problems, and keeping the networks informed of the progress of the process—the Steering Group is responsible for actively contributing to the development of the different methodological components. In particular, it provides contextual information, promotes rapprochement between networks and the incorporation of new contacts into the Network Partner System, encourages initial collective reflection within the networks, and facilitates the comparison of ideas and information.

The initial conversations held with actors from grassroots social networks in the school community, together with the information gathered from the school management team and existing formal organizations, provide the basis for beginning the study of the spatial and network structure of the educational community. The development of Transects and Drifts [43], and the creation of Network Mappings [47] and Sociograms [48,49], makes it possible, on the one hand, to understand how different groups use physical space and, on the other, to visualize the types of relationships—affinity, opposition, complementarity, alienation, among others—that they maintain with one another. This information is particularly useful for detecting possible processes of spatial appropriation [50], or places where violent practices or events linked to bullying tend to occur, as well as for recognizing conflictive situations between different actors and group configurations.

At this stage, the Direct Action for Transformative Peace strategy is implemented through the design of a communication and social mobilization plan [43] aimed at encouraging the participation of the educational community as a whole. It is particularly important to involve the Steering Group and actors linked to the Network Partner System in facilitating the design and implementation of this communication strategy.

The concluding point of this first phase is the preparation of a preliminary technical diagnosis: a document that synthesizes secondary situational and contextual information, the initial results of the study of the spatial and network structure, and the information initially gathered from both key informants and conviviality experts [33,34], as well as from the first actors contacted. For this purpose, the use of open, semi-structured, and group interviews, informal meetings, participant observation [51,52], and other information-gathering techniques, such as questionnaires, is particularly useful. This document should also serve to refine the methodological planning of the process in subsequent phases. It is advisable to develop, and later revise in a participatory manner, an Action Research Matrix [46] that brings together the general and specific cognitive and operational objectives, the techniques, sources, units of analysis, and associated indicators, as well as a detailed, phase-based work schedule.

Table 1 presents the methodological components of the first phase, together with the associated techniques, dynamics, and tools.

#### 2.1.2. Phase II: Conversations Within Networks

The second phase of the methodological process aims to foster reflection within grassroots networks to develop a participatory self-assessment that includes an analysis of the phenomenon of bullying, as well as related conflictive and problematic situations. In this phase, it is necessary to deepen the study of the spatial and network structure begun in the previous stage, through a participatory analysis that enables an understanding of the web of relationships configured around particular cases of bullying, and to proceed with the collection and sociological–discursive analysis of primary information [54]. This makes it possible to account for the arguments, reasons, and ideas on which the behaviors maintained by different groups and social networks in various cases of bullying are based. It is therefore important to identify and characterize which group realities participate in the phenomenon and how they do so and, where this corresponds to the scope initially defined, to understand the specific dynamics of each case, identifying the different elements that constitute its structure and the logic that facilitates its reproduction.

For this purpose, a participatory analysis of the phenomenon of bullying is carried out, following proposals for participatory conflict analysis and drawing on techniques such as the Conflict Analysis and Reflection Matrix [25,46], as well as other tools, including the analysis of positions, interests, and needs [55], the SWOT Matrix [56] and the DRAFPO Matrix [43], the nested problem paradigm [57], the integrated conceptual framework [40], the flowchart [58,59], and the conflict tree [60]. It is also relevant at this stage to proceed with the collection of primary information within the networks, through open and semi-structured interviews [33,34] and discussion groups [33,34,61].

During this phase, it is advisable that both the collection of primary information and the application of participatory analysis techniques be carried out separately within the different group networks, rather than concurrently, to encourage the emergence of a genuine and comprehensive discursive flow. Such a flow is essential for in-depth analysis and for its subsequent feedback in the third phase of the process. It is crucial to identify which actions are undertaken by the different group realities involved in each case of bullying, why they undertake them, for what purposes, towards whom they are directed, how and when they are carried out, and how they are justified. Given that these are potentially sensitive issues, the collection of primary information must be designed so as to minimize expressive obstacles—such as embarrassment or fear of exposure in front of certain interlocutors—thereby helping to ensure the fluidity and authenticity of the discourse.

In the second phase, the Education for Transformative Peace strategy is also continued through the design and implementation of training processes specifically tailored to the networks most closely linked to existing cases of bullying in the school. The set of training workshops—whose specific topics will be defined on the basis of the concrete features of the bullying cases and the ways in which each group reality is connected to the phenomenon—should promote the development of knowledge about bullying (its conception, characteristics, dynamics, impacts, etc.) and about how it manifests in the school. It is likewise recommended that the training processes within the networks include the study and analysis of themes drawn from Peace and Conflict Studies, such as peace theory, conflict and violence, principles of nonviolent action, and emotion management and emotional self-care. The development of participatory workshops using techniques, games, and dynamics drawn from popular education traditions [30,37], sociodrama [44,53], psychodrama [62], methodologies derived from the Theatre of the Oppressed [63,64], and film forums [65], among others, can be particularly useful tools for this purpose.

Finally, in this second phase, the Direct Action for Transformative Peace strategy is also continued through the collective definition of progressive attitudinal and behavioral changes in the different networks, promoting the implementation of informational, dissemination, and symbolic actions with those networks that show greater involvement.

Building on the initial intra-network reflections generated both in the training spaces activated during this phase and within the Steering Group and the Network Partner System, and drawing on the Outcome Mapping methodology [66,67], a series of behavioral changes are identified, made visible, and monitored. These changes are specified in concrete actions to be undertaken by the actors involved and are recorded in an Outcome Monitoring Matrix, which incorporates progress markers, intervention strategies, and evidence collected for follow-up. Initially focused on the most active and committed networks, this process is of particular strategic importance, as it lays the groundwork for facilitating inter-network dialogue in the third phase.

The preparation of the Self-Diagnosis document, which concludes this second phase, not only systematizes the information analyzed within the networks but also makes it possible to adjust the initial planning, redefining the objectives established or the proposed course of action, where necessary.

Table 2 lists the methodological components of the second phase and the associated techniques, dynamics, and tools.

#### 2.1.3. Phase III: Conversations Between Networks

In the third phase, it is important to foster second-order reflection, through which the logics underpinning the phenomenon of bullying and its associated problems are explored and questioned [35]. The aim is to debate the arguments, reasons, and ideas that sustain the production and reproduction of insults, mockery, or physical violence, as well as the indifference, rejection, or contempt experienced in the spaces shared by the actors, groups, and networks involved. The sharing of the self-diagnosis findings with the networks, in order to encourage collective deliberation among them, must take into account the capacity for objectification that every subject possesses, promoting the observation of one’s own observation and of the observations made by others, so that it becomes possible to think and speak about what has been said or is going to be said. This involves reflecting on what is said or left unsaid, from which positions, by whom, why, and for what purpose [29], to help overcome confrontations and axes of opposition, contribute to inter-network rapprochement, and support the gradual emergence of new peaceful relationships between networks.

To this end, it is necessary to promote the construction and dissemination of reversive and boundary-transcending arguments through which critical knots, opposing sets of actions, or internal resistances within groups can be unlocked [35]. The emergence and circulation of such arguments should be stimulated in the very exercises of feeding back the analyzed information, through Participatory Social Creativity Encounters and the use of techniques such as sociodramas [44,53], reflexive matrices [33], tetralemmas and discursive polyhedra [34,49,68,69], as well as problematizing questions and maieutic dialogue: the art of questioning the logic underpinning statements and facilitating the observation of contradictions [70]. These resources are conceived as a flexible methodological repertoire that can be used individually or in combination, depending on the characteristics, needs, and pace of each specific process.

In this stage, it is also necessary to continue the educational strategy, promoting theoretical and practical training on themes related to Peace and Conflict Studies, as well as on specific skills, abilities, and competences that foster pacifist empowerment [41], dialogue and understanding, respect, justice, and forgiveness among networks. The training processes in this phase could address topics such as emotional competences [71], self-knowledge and emotional self-regulation, nonviolent assertive communication [72], active listening [73], the development of emotional intelligence [74,75], the construction of cognitive and affective empathy [76], and methods of nonviolent action [38], as well as digital literacy for the prevention of cyberbullying [77]. Seminars, forums, discussions, and talks may be organized, together with training and reflection techniques and tools such as dialogue circles [78], Theatre of the Oppressed [63], case analysis, and even the practice of mindfulness in schools [79,80].

Finally, during the third phase of the process, it is appropriate to establish specific agreements for the regulation and transformation of as many cases of bullying as possible. Drawing on the Integrated Conceptual Framework [40], the Transcend Method [81], techniques such as the Conflict Reflection Matrix [46] and the aforementioned tetralemma, together with reversive and boundary-transcending arguments and the reflections and lessons learned through the educational strategy and direct transformative action, it becomes possible to deepen dialogue between networks, fostering the transformation of the network structure formed around cases of bullying. In this way, victim networks will begin to break their exclusion, benefiting from the growing engagement of networks that progressively abandon their indifference, as well as from the withdrawal of those that encourage violent actions.

At this point, some alternative dispute resolution methods can also be used as complementary tools, depending on the specific case [55,65,82,83]. However, it is important to emphasize that the proposed methodological process requires a comprehensive approach to bullying cases as a whole [42], transcending the logic of approaches that address them individually or in isolation. It is considered crucial that the management of agreements is not conceived as a product detached from the overall transformation process, but rather as fully articulated with it. Within this component, it is necessary to give continuity to the process of change in the networks by following up on the signs of progress developed through Outcome Mapping [66,67] and by configuring spaces for inter-network action with the aim of multiplying artistic and awareness-raising initiatives through socio-community animation [84].

The conclusion of this third phase is marked by the development of the Discursive Consensus Trencadis [46]. This document systematizes the scope of collective reflections and the relational and behavioral changes generated between networks, while synthesizing the status of bullying cases, the agreements reached, and the most pressing issues, demands, and needs. It also identifies, for each strategic line, the elements of consensus on which it will be possible to build, in a participatory manner, the Plan for Peaceful School Coexistence in the subsequent phase.

Table 3 lists the methodological components of the third phase and outlines the associated techniques and tools.

#### 2.1.4. Phase IV: Projective Conversations

The fourth phase aims to develop a Plan for Peaceful School Coexistence, drawing on second-order reflections, the processes of attitudinal and behavioral change, the regulation of bullying cases, and the inter-network rapprochement promoted during the previous phase. This plan must integrate the actions already underway within the three strategic lines, ensuring continuity of the processes initiated, while also incorporating new actions that specify the tasks to be implemented and the economic, human, and material resources required.

Designing this plan requires thinking simultaneously about the ideal and the possible school community. It is therefore necessary to consider existing limitations in terms of resources and capacities, the state of the whole process, and the specific situation of bullying cases. The collective definition of a horizon of transformation towards which the plan will be oriented can be particularly useful for the subsequent construction and prioritization of proposals for action. The Outcome Mapping [66] methodology offers various tools for reflecting on and defining a Vision–Mission [67], although other options may also be equally relevant. As noted above, the aim is to promote the densest, most extensive, and most intense inter-networked action collective as possible [29], intentionally articulated towards the realization of that horizon.

Collectively defining the most appropriate proposals to be developed is as important as defining the horizon itself. It is advisable to apply a series of techniques and dynamics that enable both the creative construction of proposals and their evaluation and prioritization in Participatory Social Creativity Meetings. Among others, the Programmatic Matrix [43], the 9-question technique [43], and tools derived from Participatory Rural Appraisal [86,87]—such as criteria-based evaluation matrices, the hierarchization of problems and proposals, alternative scenarios, option-selection matrices, task- and time-allocation matrices, and decision-making matrices—can be employed, together with the definition of the Key Idea [35] and weighted voting.

The collective development of school coexistence rules is also a key component of the Plan for Peaceful School Coexistence. In this regard, it is necessary to create spaces for reflection and collective rulemaking in the different grades, classrooms, and educational levels, organized into a coherent system that, taken as a whole, promotes comprehensive peaceful coexistence. The resulting regulatory system should go beyond the mere regulation of student behavior and also address the behavior of the entire educational community, the dynamics between institutional bodies, and the demands and expectations of students, teachers, and parents.

To facilitate the implementation and proper coordination of the activities included in the Plan, it is necessary throughout this fourth phase to continue the educational strategy, strengthening the networks’ capacity for management, coordination, and implementation. In this context, issues such as distributed leadership [88] and participatory leadership [89], models of school sociocracy [90], participatory organization and co-management [43], and group dynamics [91] acquire particular relevance. Likewise, training in this phase should deepen work on themes related to listening and dialogue, alternative conflict resolution, and the importance of personal and social change.

Finally, it is necessary to continue the strategy of transformative action in bullying cases and the processes of change within networks, promoting their integration into the Plan for Peaceful School Coexistence. The structure resulting from this phase will be set out in the Action Plan document, which marks the conclusion of the fourth phase of the process.

Table 4 lists the methodological components of the fourth phase and presents the associated techniques, dynamics, and tools.

#### 2.1.5. Phase V: Proactive Conversations

The fifth phase of the process involves the implementation, monitoring, and participatory evaluation of the Plan for Peaceful School Coexistence. In this phase, it is necessary to promote a participation and co-management structure for the implementation of the plan that is as horizontal and democratic as possible, bringing together the Steering Group, the Network Partner System, all the active groups formed throughout the process, the Monitoring Committee, and any strategic alliances established with local social organizations and public institutions. To ensure effective participation by the educational community networks, it may be useful to configure working teams with clearly defined functions, drawing on structures such as Mixed Working Groups and Sectoral or Thematic Tables [43]. The definition of a Coordinating and Mobilizing Team, with a non-directive facilitating role, can also help to encourage participation and effective, horizontal coordination. It is advisable that the bodies created at this stage operate under the principles of role rotation, transparency, and accountability in regular meetings, avoiding the concentration of power and promoting distributed leadership [88].

To ensure that the Plan is implemented appropriately, it is relevant to employ operational tools for participatory co-management, such as Operational Matrices [43], work schedules with clear yet flexible milestones [86], Critical Incident Matrices to record, analyze, and learn from conflict or crisis situations, and participatory indicators and sources of verification [35,43,86].

The educational strategy in this phase focuses on strengthening the structure for school participation and co-management through training in key capacities and competences for organizational development, collective action, implementation, management, and participatory monitoring of the activities of the Plan for Peaceful School Coexistence. This training is not merely technical but political–pedagogical, combining mastery of operational tools with critical reflection on power, inclusion, and democracy in the school environment. It may be useful to design role-playing sessions, assembly simulations, case studies, and Participatory Social Creativity Meetings on themes such as techniques for implementation, monitoring, and participatory evaluation, democratic decision-making mechanisms [92], horizontal group facilitation and conflict management in assemblies [93], distributed leadership [88], peace circles [78], and nonviolent dialogue and communication [72], among others.

To conclude phase five, it is necessary to systematize the experience [94] in order to reconstruct the process critically, identify good practices and lessons learned, adjust strategies, and establish improvements for its continuity. This process—carried out through collective memory workshops, participatory timelines, critical incident analysis, and dialogic evaluation among participants—will not only document achievements and challenges but also strengthen critical awareness of power, participation, and democratic coexistence in the school. The final evaluation, based on co-constructed indicators [43,86], will generate a living, shared report that will serve as a basis for the institutional continuity of the process, helping to ensure its sustainability, adaptability, and scalability in subsequent cycles. The end of the fifth phase opens onto a new beginning at a different point from Phase I, insofar as participants consider it appropriate.

Table 5 lists the methodological components of the fifth phase and presents the associated techniques, dynamics, and tools.

## 3. Discussion

Traditional definitions of bullying generally converge on three key elements—repetition, intentionality, and an imbalance of power between the parties—and tend to frame it primarily as a dyadic dynamic between victim and aggressor [4,5,6]. However, bullying is a multi-causal phenomenon, articulated through differentiated group roles [9] and conditioned by factors such as classroom climate, parenting styles, and the specific characteristics of each context [8,10,11]. It is also embedded in broader configurations of inequality and in cultural and structural forms of violence [12,13,14,15]. This relational conception is consistent with the notion of the total social fact [42]; yet methodological devices that enable the systematic involvement of the different school networks in processes for the prevention and mitigation of bullying remain insufficiently developed and scarcely disseminated.

Research on bullying has largely focused on estimating prevalence and identifying individual characteristics as primary causes of the phenomenon [1,3], thereby reinforcing adult-centric perspectives [16] and limiting understanding of how bullying unfolds in specific contexts. Recent participatory experiences show important advances; however, they tend to concentrate on specific groups of actors (either students, parents, teachers, etc.) and to describe relatively limited exercises of consultation or participation [4,10,18,19,20,21,22]. These initiatives do not necessarily address bullying as a socially constructed reality that requires reconfiguring the relational structures that sustain it, nor do they fully consider all the networks involved in the production, normalization, or tolerance of violence.

The Method for Participatory Construction of Peaceful Coexistence [24,25,26], grounded in the sociopraxical perspective of Transformative Peace [27,28,29] and the Freirean perspective [30,37], proposes a spiral sequence of action–reflection–action that, through five phases of intra- and inter-network conversations, facilitates the transformation of the school community’s ways of feeling and thinking [31] about bullying. The collective process interrogates the culture of violence and the construction of identities and belongings, through the development of a diagnosis and an intervention in which the entire network system within the school environment participates.

In the first phase, Initial Conversations, the methodological development of the approach is planned and its organizational structure defined, through a set of components that anchor the process in its specific institutional and sociocultural context. The second phase, Conversations within Networks, seeks to promote reflection within grassroots networks in order to build a participatory self-diagnosis that includes an analysis of bullying and of the associated conflictive and problematic situations. In the third phase, Conversations between Networks, the focus shifts to inter-network dialogue: the information generated in the previous stages is collectively revisited to foster second-order reflection on the arguments, positions, and meanings that sustain bullying dynamics. The fourth phase, Projective Conversations, is dedicated to the collective design of a Plan for Peaceful School Coexistence, drawing on these second-order reflections, on the attitudinal and behavioral changes initiated, on the agreements reached regarding bullying cases, and on the inter-network rapprochement promoted in the preceding phase. Finally, the fifth phase, Proactive Conversations, centers on the implementation, participatory monitoring, and evaluation of the Plan for Peaceful School Coexistence.

These five phases and the associated components and techniques presented do not operate as a rigid script that predetermines the process, but as an orienting framework that must be continuously reworked with participants. The concrete configuration of each phase, the selection and combination of techniques, and even the sequencing of activities are to be defined and adjusted collectively in each context; the list of methodological tools is to be understood as a repertoire from which actors choose and adapt options according to their needs, rather than as a mandatory checklist; and the strategy is intentionally open to being reshaped by emerging questions, unexpected dynamics, and new forms of collective agency that appear throughout the process. In this way, the proposal seeks to reconcile the need for a structured, systematized approach—which makes processes transferable and evaluable—with the openness, flexibility, and ongoing re-elaboration that are constitutive of participatory research and generate sustainable transformations in bullying dynamics.

Issues of time, resources and institutional buy-in are central to the feasibility of this proposal. The method does not presuppose unlimited availability, but is grounded in more than two decades of sociopraxic participatory work carried out in collaboration with public institutions. In these experiences, the only direct financial cost has been the external facilitation/research team, while all other actors participate as part of their ordinary functions. Typical budgets for local participatory processes have remained at levels that are generally manageable for municipal or regional administrations and, where necessary, can be shared across clusters of institutions rather than assumed by a single center. Moreover, the methodological process is conceived as a spiral and is strongly context-dependent: some experiences have lasted only a few months, whereas others have extended over longer periods, with phases adapted to institutional rhythms and participation and shared ownership built progressively over time.

Starting from the premise that bullying violence rests on the non-recognition of the other, the methodological proposal goes beyond the limits of sanctions and mediation. Its purpose is to deactivate the bases that sustain those who bully by fostering an opinion climate that is favorable to diversity and critical of exclusionary models. The combination of Research for Transformative Peace [32,33,34,35], Education for Transformative Peace [26], and Direct Action for Transformative Peace enables the development of strategies aimed at reconfiguring networks and meanings in ways that transform the phenomenon of bullying.

This theoretical–methodological proposal has concrete implications for school policies. It questions the centrality of regulations and sanctions as the main tools for preventing bullying, recognizing that their impact is limited and that they may generate reprisals and reinforce symbolic violence against victims. In contrast, it advances the participatory design of School Coexistence Plans that involve the educational community, operating on the networks, norms, and shared understandings that make bullying possible. Implementing this process requires specific institutional and community conditions, including openness on the part of school leadership and management, material resources, and a facilitation team capable of sustaining participatory processes over time.

It is also important to acknowledge that participatory processes may reveal aspects of school life that are uncomfortable for the commissioning institution, leadership teams, or other actors. From the outset, it is therefore essential to assume that all parties are simultaneously subjects and objects of knowledge production and of the proposals for action that emerge. The process entails sustained self-critique and reflexive examination of what is said and done, how it is said and done, and with what effects. This implies that school authorities, staff, students, and families must be willing to question their own practices, assumptions, and forms of participation in the dynamics that make bullying possible. If this initial premise of shared reflexivity and co-responsibility is not accepted, the process runs the risk of becoming blocked, reduced to a merely procedural exercise, or confined to superficial changes that do not alter the underlying relational structures.

## 4. Future Directions

Future research should undertake comparative studies of rigorously systematized empirical processes of Participatory Construction of Peaceful Coexistence to examine both their concrete outcomes in terms of reducing or transforming bullying and the specific configurations these participatory processes adopt in different contexts. Such comparative work would make it possible to generate evidence on the enabling and constraining factors for the peaceful transformation of bullying in diverse socio-political–economic–cultural settings, to identify patterns or typologies in bullying dynamics, to compare evidence-based good practices, and to delineate structural, institutional, and cultural factors that either reproduce violent dynamics or hinder the deployment of effective participatory processes. It would also allow a more precise assessment of the relevance and transformative potential of the various methodological components of the Method for the Participatory Construction of Peaceful Coexistence, refining the design of School Coexistence Plans. Longitudinal research that combines social network analysis, discursive approaches, and process-tracing of school communities over time is particularly needed to understand in which contexts, and under what conditions, the methodology generates sustained transformations in bullying dynamics.

A second line of future work will focus on an ethnomethodology of conflict consistent with the sociopraxic perspective adopted in this article. If conflict, like any social reality, is socially constructed, it is essential to examine how actors infer meaning from their experiences and how collectively shared representations organize, classify, and stabilize social realities. This requires promoting systematic reflection on the discourses of those involved in conflict: not only on what is said, but on when, why, by whom, for what purpose, and from which positionalities it is said. The epistemological and theoretical justification for fostering this reflexivity will be the focus of forthcoming work, with the aim of strengthening both the analytical and mediational capacities of participatory approaches to bullying and other school conflicts.

## Figures and Tables

**Table 1:** Strategic lines, methodological components, techniques and tools associated with Phase I: Initial conversations.

Strategic Line	Methodological Components	Techniques or Tools
Starting Point 1	Formation of the technical team and initial self-reflection	Initial Self-Reflection Matrix: questions and first ideas [43] Sociodrama [44,53]
	Establishment of the formal relational framework with the management team and formal organizations of the educational community, entities, and institutions involved	Initial Negotiation [43] or Demand Negotiation [45] Trust Agreement [46] Monitoring Committee [43,45]
Education for Transformative Peace	Educational strategy (I): self-training of the technical team for the development of competences appropriate to the context	Seminars, workshops, forums, symposia, dialogic spaces data
Research for Transformative Peace	Arrival and integration into the educational community: identification and establishment of the relational framework with actors, groups, and networks of students, teachers, and parents’ data	Network Partner System [46] Steering Group [42,44]
	Study of the space and reticular structure of the educational community (I)	Transects and Drifts [43] Network Mapping and Sociogram [47,48,49]
	Collection and analysis of information (I): secondary information and primary information from initial conversations	Secondary information: Sociodemographic, socioeconomic, and socio-educational characteristics [33,34] of the educational community and its sociocultural environment. Open and semi-structured interviews with key informants and coexistence experts [33,34] Primary information: Participant Observation [51,52] Working and follow-up meetings of the Steering Group, Network Partner System, and Monitoring Committee Informal encounters; open, semi-structured, and group interviews; and Discussion Groups [33,34] Questionnaires
Direct Action for Transformative Peace	Transformative Action Strategy (I): expansion of the process to new networks	Communication Plan [43]
Closing or Conclusion Point 2	Preparation of the technical pre-diagnosis document and definition of methodological planning	Systematization of primary and secondary information Action Research Matrix [46]

**Table 2:** Strategic lines, methodological components, techniques and tools associated with Phase I: Conversation within networks.

Strategic Line	Methodological Components	Techniques or Tools
Research for Transformative Peace	Study of the space and reticular structure of the educational community (II): analysis of the network fabric in cases of bullying	Participatory analysis of the space and network structure of bullying cases: Transects and Drifts [55] Participatory Network Mapping and Sociograms [47,48,49] Flowchart [58,59] Conflict Analysis and Reflection Matrix [46] Nested Problem Paradigm [57] Integrated Conceptual Framework [40] SWOT Matrix [56] and DRAFPO Matrix [43] Conflict tree [60] Analysis of positions, interests, and needs [55]
	Collection and analysis of information (II): primary information within the networks	Collection and sociological–discursive analysis [54] of primary information in the networks: Participant Observation [51,52] Open, semi-structured, and group interviews, and Discussion Groups [33,34,61]
Education for Transformative Peace	Educational strategy (II): self-reflection within the networks and initial theoretical–practical training	Self-reflection and training in Participatory Meetings of Social Reflection developed through games, dynamics, and techniques of Education for Peace: Initial Self-Reflection Matrix [43] Sociodrama [44,45,46,47,48,49,50,51,52,53] Psychodrama [62] Theatre of the Oppressed: Games, Forum Theatre, Image Theatre [63,64] Film forum [65]
Direct Action for Transformative Peace	Transformative Action Strategy (II): definition of change processes in the networks	Outcome Mapping [66,67] Participatory Meetings of Social Creativity for the design and development of transformative actions
Closing or Conclusion Point 3	Preparation of the Self-Diagnosis document	Systematization of the information and reconfiguration of the Research Matrix, schedule, and methodological strategy.

**Table 3:** Strategic lines, methodological components, techniques and tools associated with Phase I: Conversation between networks.

Strategic Line	Methodological Components	Techniques or Tools
Research for Transformative Peace	Feedback of the Self-Diagnosis: second order feeling–thinking reflexivity between networks and construction and dissemination of reversive–boundary-transcending arguments	Feedback of the analyzed information in Participatory Meetings of Social Creativity to promote second-order collective reflexivity and inter-network rapprochement and dialogue: Reflexive Matrix [33] Tetralemma/Discursive Polyhedra [34,49,68,69] Problematizing Questions and Maieutic Dialogue [70] Sociodrama [44,53] Nested Problem Paradigm [57] Integrated Conceptual Framework [40] SWOT Matrix [56] and DRAFPO Matrix [43] Conflict tree [60] Analysis of positions, interests, and needs [63]
Education for Transformative Peace	Educational strategy (III): critical analysis and pacifist empowerment of networks	Theoretical–practical training through Participatory Group Reflection Meetings, Dialogue Circles [78], seminars, forums, discussion spaces, talks, Theatre of the Oppressed [63], and school-based mindfulness [79,80], on topics associated with Peace and Conflict Studies and specialized training in specific capacities, abilities, skills, and competences: Emotional intelligence [74,75] and emotional competencies [71,85] Nonviolent assertive communication [72] and active listening [73] Cognitive and affective empathy [76] Digital literacy on cyberbullying [77] Methods of nonviolent action [38]
Direct Action for Transformative Peace	Transformative Action Strategy (III): regulation of bullying cases and continuity of change processes in the networks	Alternative dispute resolution methods [55,65,82,83] Integrated Conceptual Framework [40] Transcend Method [81] Conflict Reflection Matrix [46] Tetralemma/Discursive Polyhedra [34,49,68,69] Continuity of change processes in the networks: Progress markers in Outcome Mapping [66,67] Multiplication of transformative actions through socio-community animation [84]
Closing or Conclusion Point 4	Preparation of the Discursive Consensus Mosaic document	Systematization of the reflections promoted, the reversive–boundary-transcending arguments between networks, and synthesis of the agreements reached for the regulation of bullying cases and by lines of work/intervention.

**Table 4:** Strategic lines, methodological components, techniques and tools associated with Phase I: Projective Conversations.

Strategic Line	Methodological Components	Techniques or Tools
Research for Transformative Peace	Development of the Plan for Peaceful School Coexistence: construction, prioritization, and planning of peace-oriented actions by strategic line, as well as school coexistence rules	Collective construction, prioritization, and planning of action proposals and school coexistence rules in Participatory Social Creativity Meetings: Outcome Mapping [66,67] Vision–Mission [67] 9-Question Technique [43] Matrices for the construction, assessment, and prioritization of proposals [86,87] Programmatic Matrix [43] Definition of the Key Idea [35]
Education for Transformative Peace	Educational strategy (IV): critical analysis and pacifist empowerment of networks for collective action	Theoretical–practical training through Participatory Group Reflection Meetings to strengthen the networks’ capacity for management, coordination, and implementation: Distributed Leadership [88] and Student Voice and Participatory Leadership [89] School sociocracy models [90] Participatory organization and co-management [43] Group dynamics [91] Nonviolent assertive communication [72] and active listening [73] Cognitive and affective empathy [76] training through Participatory Group Reflection Meetings, Dialogue Circles [78], seminars, forums, discussion spaces, talks, Theatre of the Oppressed [63], and school-based mindfulness [79,80] on topics associated with Peace and Conflict Studies and specialized training in specific capacities, abilities, skills, and competences: Emotional intelligence [74,75] and emotional competencies [71,85] Nonviolent assertive communication [72] and active listening [73] Cognitive and affective empathy [76]
Direct Action for Transformative Peace	Transformative Action Strategy (IV): continuity of the processes of regulating bullying cases and change within networks	Alternative dispute resolution methods [55,65,82,83] Integrated Conceptual Framework [40] Transcend Method [81] Conflict Reflection Matrix [46] Tetralemma/Discursive Polyhedra [34,49,68,69] Continuity of change processes in the networks: Progress markers in Outcome Mapping [66,67] Multiplication of transformative actions through socio-community animation [84]
Closing or Conclusion Point 5	Preparation of the Plan for Peaceful School Coexistence document	Systematization of proposals for transformation by lines of work/intervention.

**Table 5:** Strategic lines, methodological components, techniques and tools associated with Phase I: Proactive Conversations.

Strategic Line	Methodological Components	Techniques or Tools
Direct action for Transformative Peace	Transformative Action Strategy (V): implementation, monitoring, and evaluation of the Plan for Peaceful School Coexistence	Tools for the implementation and co-management of activities: Operational Matrices [43] Work schedules [86] Critical Incident Matrix, Indicators and Sources of Verification [35,43,86] Matrices for participatory monitoring and evaluation [43,86]
	Structure for participation and co-management of the Plan for Peaceful School Coexistence	Articulation of the Steering Group, the Network Partner System, the Monitoring Committee, strategic alliances, and the formation of: Mixed Working Groups [43] Sectoral/Thematic/Geographical Tables [43] Coordinating and Mobilizing Team
Education for Transformative Peace	Educational strategy (V): critical analysis and pacifist empowerment of the participation and co-management structure of the Plan for Peaceful School Coexistence	Theoretical–practical training through Participatory Group Creativity Meetings on themes associated with Peace and Conflict Studies and specialized training in specific capacities, abilities, skills, and competences for organizational strengthening, collective action, and mediation: Techniques for implementation, monitoring, and participatory evaluation: Operational matrices, work schedules, and indicators with sources of verification; Democratic decision-making mechanisms [92] Horizontal group facilitation and conflict management in assemblies [93] Distributed leadership [88] Peace circles [78] Nonviolent dialogue and communication [72] Participatory management, group dynamics, and leadership [91]
Closing or Conclusion Point 6	Preparation of the Monitoring and Evaluation document for the Plan for Peaceful School Coexistence	Participatory systematization of the existence [94]

## Data Availability

Not applicable.
